# Protective effect of Curcumin on chronic cerebral ischemia by altering expression of α- synuclein in 2VO model

**DOI:** 10.1186/1750-1326-7-S1-S33

**Published:** 2012-02-07

**Authors:** Gang Yu, Li Liu, Peng Zhang, Yu Li

**Affiliations:** 1 Department of Pathology, Chongqing Medical University, China; 2 Institute of Neuroscience, Chongqing Medical University, China; 3 Chongqing Key Laboratory of Neurobiology, Chongqing Medical University, China; 4 Department of Neurology, The First Affiliated Hospital, Chongqing Medical University, Chongqing, 400016, China

## Background

Previous studies have shown that natural compound Curcumin can improve some biological effects induced by chronic cerebral hypoperfusion and may represent a target for treatment. α- synuclein oligomerization and aggregation are considered to have a role in the pathogenesis of brain ischemia/reperfusion. However, the effects of Curcumin on α- synuclein in chronic cerebral hypoperfusion are poorly understood. This study aims to observe the effect of Curcumin on chronic cerebral ischemia model in rats and investigate change of α- synuclein induced by Curcumin.

## Methods

The chronic cerebral ischemia was produced in male Sprague-Dawley rats by permanent occlusion of bilateral common carotid arteries (2VO). Animals were randomly divided into 5 groups: normal control group, sham-operated group, 2VO+DMSO group, 2VO+Curcumin 100mg/kg group, 2VO+Curcumin 50mg/kg group. After surgery, all animals were injected intraperitoneally with DMSO solution of Curcumin or a same volume of normal DMSO. Each group was injected once daily for four consecutive weeks. After the completion of the behavioral testing, rats were sacrificed. Hematoxylin-eosin staining and Nissl staining were carried out in section. The expressions of α- synuclein protein in hippocampus were detected by immunohistochemistry.

## Results

The chronic cerebral ischemia in rats resulted in a significant pathological change in the hippocampus CA1 area, including: loss of pyramidal cell, shrinkage of nuclei, dark staining of neurons, loss of Nissl body and glial proliferation as compared with sham-operated rats. The administration of different doses of Curcumin attenuated neuronal injury in rats induced by chronic cerebral ischemia along with the concomitant increased the numbers of α-synuclein -positive cells.

## Conclusion

Our data demonstrated that the neuroprotective effect of Curcumin involved in increasing the protein levels of α-synuclein induced by ischemia.

